# Perturbation of periodic spot-generation balance leads to diversified pigmentation patterning of harlequin *Phalaenopsis* orchids: in silico prediction

**DOI:** 10.1186/s12870-024-05305-z

**Published:** 2024-07-18

**Authors:** Ti-Wen Lu, Wen-Huei Chen, Pao-Yang Chen, Yu-Chen Shu, Hong-Hwa Chen

**Affiliations:** 1 Department of Life Sciences, National Cheng Kung University, Tainan, 701 Taiwan; 2 Orchid Research and Development Center, National Cheng Kung University, Tainan, 701 Taiwan; 3 Institute of Plant and Microbial Biology, Academia Sinica, Taipei, 115 Taiwan; 4 Department of Mathematics, National Cheng Kung University, Tainan, 701 Taiwan

**Keywords:** Harlequin *Phalaenopsis*, Retrotransposon *HORT1*, MicroRNA (miRNA), Pigmentation patterning, Turing patterns

## Abstract

**Background:**

A retrotransposon *HORT1* in the promoter of the anthocyanin activator gene *PeMYB11*, microRNA858 (miR858) that targets *PeMYB11*, and a repressor *PeMYBx* have been implicated in pigmentation patterning diversity of harlequin *Phalaenopsis* orchids. However, the interrelationship among them remains to be elucidated.

**Results:**

To understand how these factors interact to generate anthocyanin spots in *Phalaenopsis*, we successfully developed a mathematical model based on the known reaction–diffusion system to simulate their interplay and refined the conceptual biological model. Intriguingly, the expression of both *PeMYBx* and *PeMYB11* were in phase for purple spot formation, even though they showed adverse effects on anthocyanin accumulations. An increase in the self-activation rate of *PeMYB11* resulted in the increased size of purple spots, but no effects on spot fusion. Decreased degradation rate of miR858 in the purple regions, led to disruption of the formation of spotted pigmentation patterning and a full-red pigmentation pattern. Significantly, the reduced miR858 level promotes the fusion of large dark purple dots induced by the solo-LTR of *HORT1*, eventually generating the purple patches. In addition, the spatially heterogeneous insertion of *HORT1* caused by the remnant solo-LTR of HORT1 derived from random homologous unequal recombination of *HORT1* in individual cells of floral organs could explain the diverse pigmentation patterning of harlequin *Phalaenopsis*.

**Conclusions:**

This devised model explains how *HORT1* and miR858 regulate the formation of the pigmentation patterning and holds great promise for developing efficient and innovative approaches to breeding harlequin *Phalaenopsis* orchids.

**Supplementary Information:**

The online version contains supplementary material available at 10.1186/s12870-024-05305-z.

## Introduction

**Fig. 1:**
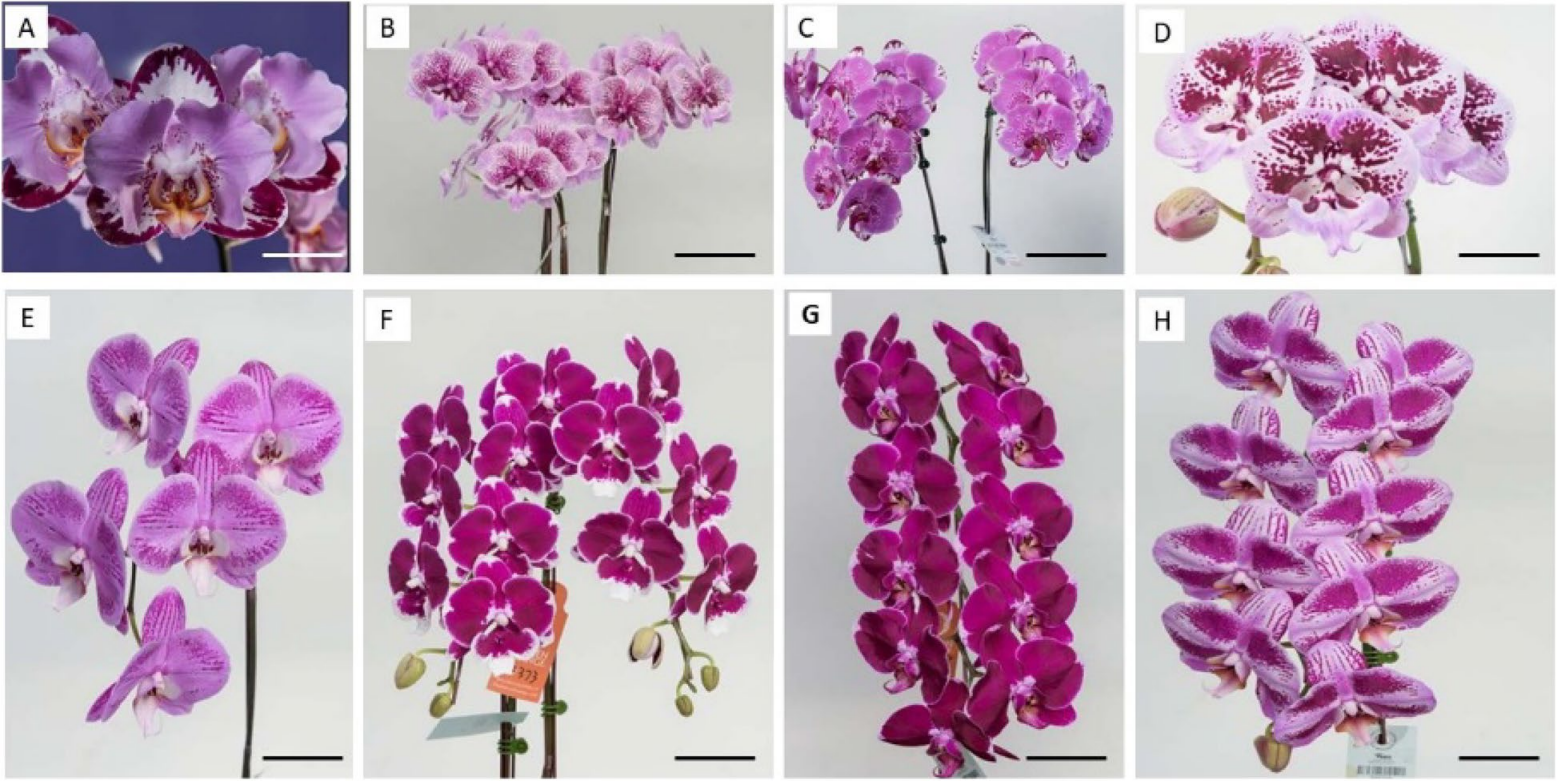
Diverse pigmentation patterns in harlequin *Phalaenopsis* orchids. **A** *P.* hybrid ‘FM1552’, Bar = 3.8 cm, **B** *P.* hybrid ‘F6965’, Bar = 8.8 cm, **C** *P.* OX Red Lion ‘OX1668’, Bar = 11.5 cm, **D** *P.* hybrid ‘OX3267’, Bar = 4.3 cm, **E** *P.* Fangmei Black Rose ‘FM1017’, Bar = 5.3 cm, **F** *P.* hybrid ‘F5373’, Bar = 7.3 cm, **G** *P.* hybrid ‘F4528’, Bar = 7.8 cm, and **H** *P.* hybrid ‘F5521’, Bar = 5.2 cm

Harlequin *Phalaenopsis* orchids, named after harlequin patches of a dark reddish-purple color, are popular because of their exotic flower shapes and diverse pigmentation patterning [1]. Some hybrids have patterning very similar to full-red patterning, whereas others have extraordinarily large spots that are not fused (Fig. 1). The dark reddish-purple coloring on the flowers results from high anthocyanin content [2]. The classical harlequin patches are related to insertion of solo-LTR but not to the full-length coding sequence of the *harlequin orchid retrotransposon 1* (*HORT1*) in the upstream promoter of *PeMYB11*. For example, *HORT1* is responsible for the spotted pigmentation patterns in the harlequin *Phalaenopsis* orchids [2]. Although *HORT1* fragments were found in white and purple regions, only the solo-LTR of *HORT1* exists in purple regions. Hence, the solo-LTR of *HORT1* appears to serve as an enhancer of *PeMYB11* and may lead to the formation of dark reddish-purple patches in harlequin *Phalaenopsis* [2]. However, *P*. Ever-spring Prince ‘Plum’ (Supplementary Fig. 1), a harlequin *Phalaenopsis* hybrid, has large purple patches in the absence of the solo-LTR of *HORT1*, and only the full-length *HORT1* was identified in *P.* Ever-spring Prince ‘Plum’ [2]. Therefore, the mechanisms of the diversified pigmentation patterning of harlequin *Phalaenopsis* remain to be elucidated.

In plants, myeloblastosis (MYB) transcription factors that serve as activators for anthocyanin biosynthesis are of the R2R3 type [3]. The R2R3-MYB transcription factor probably activates anthocyanin biosynthesis by directly binding to the promoters of structural genes for anthocyanin biosynthesis [3]. Antagonistic repression of anthocyanin accumulation by repressors of R2R3-MYB or R3-MYB also occurs in the regulation of anthocyanin biosynthesis [3]. These MYB-associated repressors can be self-activated or activated by the R2R3-MYB-bZIP-WD40 (MBW) activation complex. The inhibitory mechanisms of the MYB repressors are classified into three proposed models: 1) self-activated MYB repressors that bind directly to the promoters of structural genes to inhibit the transcription; 2) MYB repressors activated by the MBW activation complex, which further compete for the same binding sites of MYB activators to reduce anthocyanin biosynthesis gene expression; and 3) R3-MYB repressors activated by the MBW complex to inhibit the activation of *bHLH* genes thus repressing the continuous formation of the MBW activation complex [3].

We have shown that *PeMYB11*, an R2R3-type transcription activator for anthocyanin biosynthesis, is responsible for spotted pigmentation patterning in *Phalaenopsis* [2, 4]. A phylogenetic tree of R2R3-MYB transcription factors in *Phalaenopsis* orchids derived from the amino acid sequences of the R2R3 region suggest that *PeMYB4*, *PeMYB5*, *PeMYB6*, *PeMYB7*, and *PeMYB8* are R2R3-type transcription repressors in *Phalaenopsis* orchids. The four genes (*PeMYB4*, *PeMYB5*, *PeMYB6*, and *PeMYB8*) are highly expressed in white regions, while *PeMYB7* has similar expression patterns in both purple and white regions [2]. Intriguingly, an R3-type transcription repressor, *PeMYBx* is highly expressed in purple regions, with about a 97-fold increase in expression [2]. In addition to these repressors, epigenetic factors such as microRNA156 (miR156) and miR858 are enriched in the white region of harlequin *P.* ‘Panda’, whose targets are *PeMYB7* and *PeMYB11*, respectively [5]. Since *PeMYB7* has similar expression patterns in both purple and white regions, we therefore focused our further study only on the effect of miR858.

Owing to the complex regulatory network of pigmentation patterning in *Phalaenopsis*, such as full red spots and venation [4], and the commercial breeding history of harlequin *Phalaenopsis* [1], the selection of suitable and representative plant material is challenging. To simplify the biological investigation of the diversified pigmentation patterning of harlequin *Phalaenopsis* into a concise and theoretical model, we turned to mathematical simulation with the theory of reaction–diffusion systems. The reaction–diffusion systems describe the principles of natural pattern formation such as spots and stripes [6]. With the reaction among various morphogens and the diffusion of morphogens in a tissue, the main feature of complex morphogenesis can be characterized. Various reaction–diffusion systems have been built by different interaction rules, and the simplest one is the activator-inhibitor system, which includes merely two components, initially described by Turing [7]. The system used in our research satisfies the following interaction rules: 1) the activator is self-activated, 2) the inhibitor is diffusible and is activated by the activator; and 3) the inhibitor represses the self-activation of the activator. Even though Turing was the first person mentioning this model, we adopted the mathematical equations proposed by Gierer and Meihardt [6, 8], which are more biochemically truthful. In this study, using the theoretical model and mathematical simulation coupled with our previous experimental data we explained how interplays of PeMYBx, full-length *HORT1*, the solo-LTR of *HORT1*, and miR858 contributed to the steady-state accumulation of PeMYB11 and resulted in the varied spotted pigmentation patterning, including distinct spots, fused spots, and full-red-like patterns in harlequin *Phalaenopsis*.

## Results

### PeMYBx is the negative regulator of anthocyanin spot patterning in *Phalaenopsis*

**Fig. 2:**
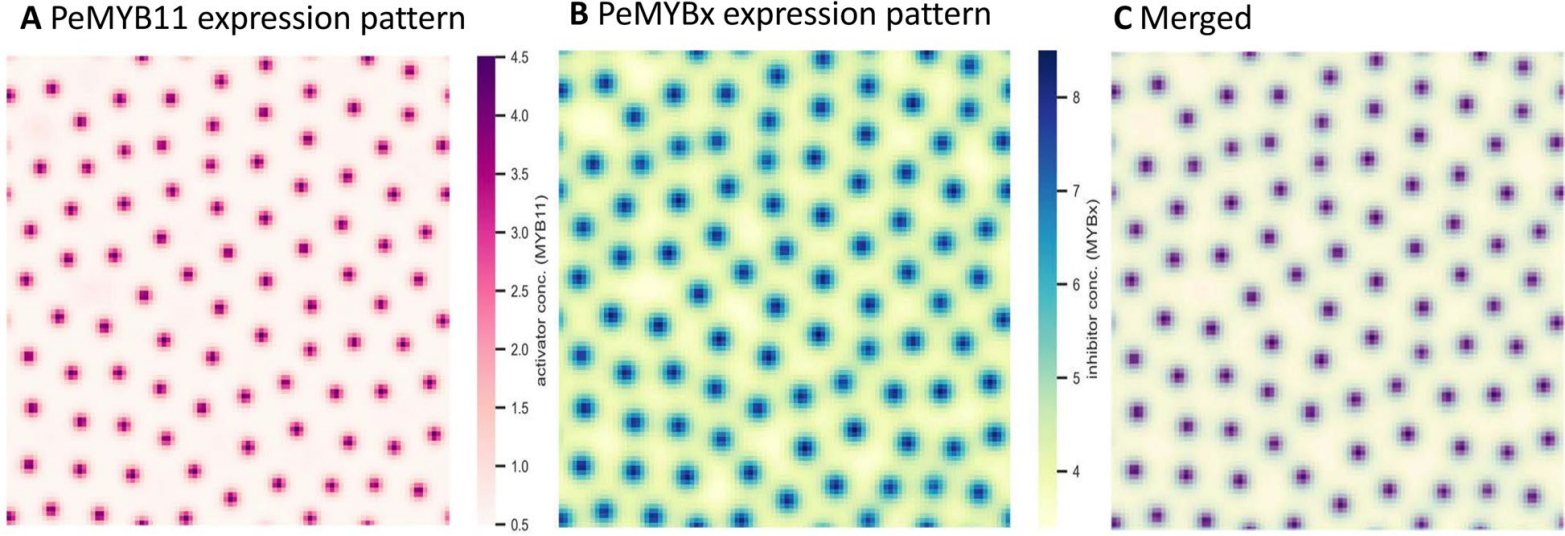
No phase difference between numerical simulations of PeMYB11 and PeMYBx expression pattern. **A** Simulated expression pattern of PeMYB11 with the following parameter values: ${D}_{A}=0.01;{ D}_{H}=0.5; {\mu }_{A}=0.03;{ \mu }_{H}=0.03;{\rho }_{A}=0.01;{ G}_{A}=0.08;{ G}_{H}=0.12$ **B** Simulated expression pattern of PeMYBx with the same parameter values as (**A**). **C** Merged

**Fig. 3:**
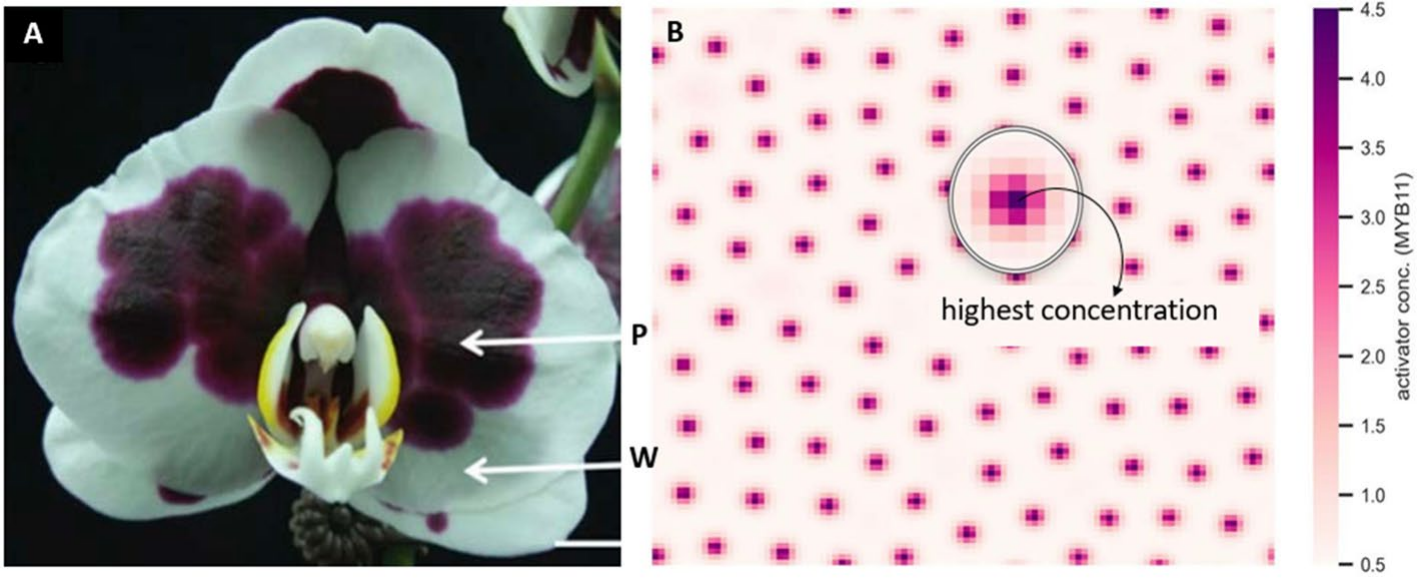
Redefinition of anthocyanin “spots”. **A** *P*. Yushan Little Pearl, P: purple region, W: white region, Bar = 1 cm. **B** The concentration of *PeMYB11* in each spot starts with the highest value and decays away from the center. Values for parameters are the same as in Fig. 4

A revisit of the mechanisms of spotted pigmentation patterning formation was essential for explaining the formation of big dark purple patches in harlequin *Phalaenopsis* cultivars. The numerical simulation showed standard spotted patterning (Fig. 2), which confirmed that the parameter values determined for the numerical simulation were suitable for the VSIMEX method used by rdsolver. Moreover, the simulations suggested that the expression patterns of *PeMYB11* and *PeMYBx* were in-phase, showing the same results as the experimental expression data for *PeMYB11* and *PeMYBx* [2]. Consequently, the PeMYB11-PeMYBx interaction model gave a preliminary explanation of why the expression of *PeMYBx* was higher in the purple than white regions. That is, instead of simply competing for the same binding sites as other MYB repressors, the diffusible repressor system of PeMYBx was more likely to inhibit anthocyanin accumulation by repressing the activator system of PeMYB11. Because of the diffusivity of PeMYBx, according to the local self-activation and lateral inhibition model, an anthocyanin spot showed the highest anthocyanin level at the center and the concentration gradually decayed out to the boundary of the spot (Fig. 3). Therefore, the purple patches of *P.* Yushan Little Pearl may be generated by anthocyanin spot fusion. In contrast, the anthocyanin level in the purple patches of *P.* Ever Spring Prince ‘Plum’ are uniformly distributed throughout the flower (Supplementary Fig. 1). Combined with the fact that the solo-LTR of *HORT1* is located only within the purple patches of *P*. Yushan Little Pearl but not *P*. Ever Spring Prince, in the following sections, we reexamined the underlying mechanisms of how the solo-LTR of *HORT1* contributes to the purple patches.

### Solo-LTR of *HORT1* increases the size of each spot of the spotted patterning of *Phalaenopsis*

**Fig. 4:**
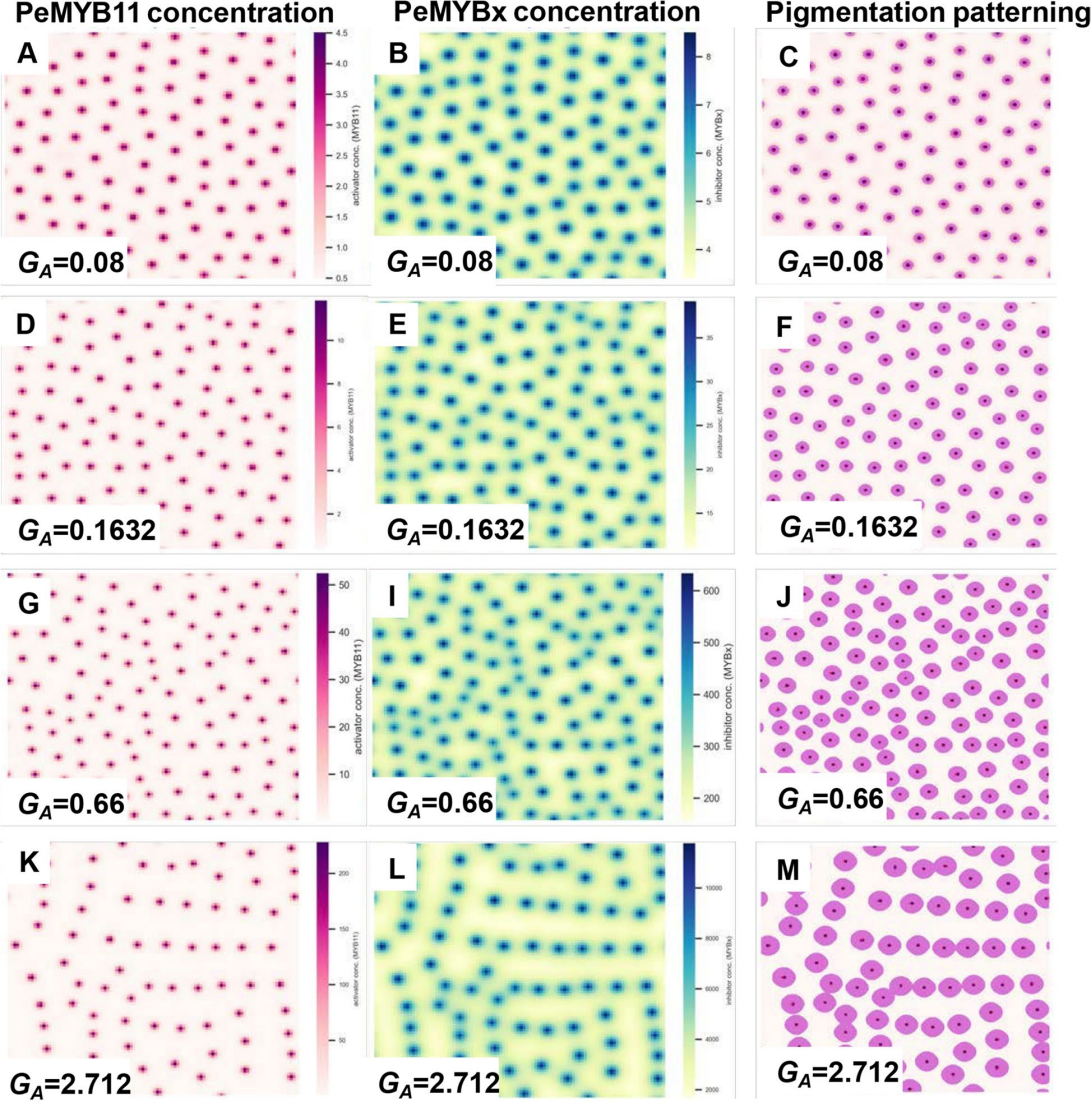
Increase in spot size proportional to the value of *G*_*A*_. **A**–**C** resemble the standard spotted pigmentation patterning with the following parameter values: ${G}_{A}=0.08,{G}_{H}=0.12,{D}_{A}=0.01,{D}_{H}=0.5,{\mu }_{A}=0.03,{\mu }_{H}=0.03, {\rho }_{A}=0.01$. **C** is generated by drawing contour maps of (**A**) at the threshold of 2 $\mu M$*.* **D**–**F** ${G}_{A}=0.1632$ is the twofold increase of the standard ${G}_{A}$ value 0.08. **F** is generated by drawing contour maps of (**D**) at the threshold of 2 $\mu M$. **G**, **I**, **J** ${G}_{A}=0.66$ is the fourfold increase of the ${G}_{A}$ value 0.1632 because harlequin-type cultivars are tetraploid plants. **J** is generated by plotting contour maps of **G** at the threshold of 2 $\mu M$. **K**–**M** ${G}_{A}=2.712$ is the 33.9-fold increase of the standard ${G}_{A}$ value 0.08 according to the data for *PeMYB11* mRNA expression in the purple regions of the flower of *P*. Yushan Little Pearl. **M** is generated by plotting contour maps of (**K**) at the threshold of 2 $\mu M$

**Fig. 5:**
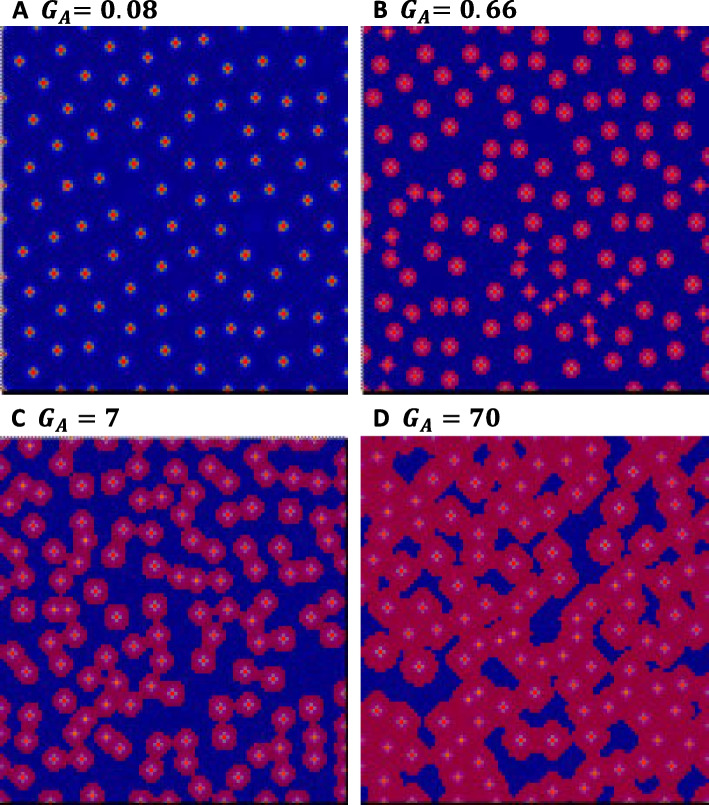
Simulation results by using the Virtual Cell. **A** ${G}_{A}=0.08$. **B** ${G}_{A}=0.66$. **C** ${G}_{A}=7$. **D** ${G}_{A}=70$. Values for other parameters are the same as in Fig. 4

Insertion of the solo-LTR of *HORT1* in the *PeMYB11* promoter increases promoter activity twofold (2). The twofold increase of the self-activation rate ${G}_{A}$, analogous to the increased transcription activity of *PeMYB11,* resulted in a slight increase in spot sizes in the simulation (Fig. 4F). More simulation results demonstrated that the simulated level of PeMYB11 increased as ${G}_{A}$ was increased and resulted in gradually increased spot sizes (Fig. 4). For validation, simulations were also implemented using Virtual Cell, modifying the model constructed by Ding et al. (2020). The values of ${G}_{A}$ in the simulations by using Virtual Cell were 0.66, 7, and 70. The simulations showed that the spot sizes increased as the ${G}_{A}$ value increased. However, even when ${G}_{A}$ was increased to 70 $\upmu M$, fusion of the spots was not increased to the level expected in *P*. Yushan Little Pearl (Fig. 5). Moreover, the center region of the spots with the highest anthocyanin accumulation did not expand to the level of the anthocyanin spots in *P.* Yushan Little Pearl (Fig. 5). The insertion of solo-LTR of *HORT1* in the promoter of *PeMYB11* could result in the big dark purple spots, but the effect on spot fusion was limited. Thus, other factors needed to be investigated to understand the exotic patterns of the harlequin *Phalaenopsis*.

### Differential miR858 expression disrupts the formation of spotted patterning of *Phalaenopsis*

**Fig. 6:**
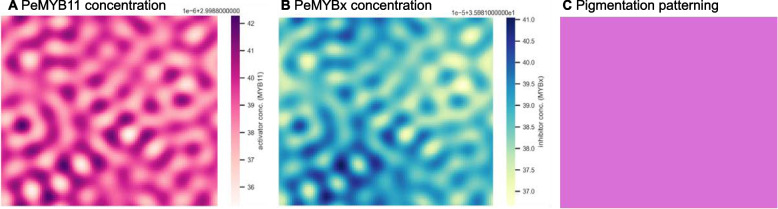
Reduction of miR858 expression disrupts the generation of spotted pigmentation patterning with an adjustment of the value of ${\mu }_{A}$ to 0.01. Values for other parameters are the same as in Fig. 4. **A** Simulated expression of PeMYB11. **B** Simulated expression of PeMYBx. **C** is generated by plotting the contour maps of (**A**) at the threshold of 2 $\mu M$

A miRNA regulates the gene expression of its targeted mRNA by degradation or inhibition of translation [12]. In harlequin *Phalaenopsis*, miR858 targeting the mRNA of *PeMYB11* is related to the formation of purple spots [8], and miR858 expression is about threefold higher in the white than purple regions [8], which suggests that the degradation rate of *PeMYB11* mRNA is lower in the purple than white regions. Thus, we assessed the role of miR858 in generating dark purple patches on harlequin *Phalaenopsis* flowers (Fig. 6). The simulation results showed that decreased degradation rate ${\mu }_{A}$, analogous to the reduced miR858 expression in the purple regions, led to disruption of the formation of spotted pigmentation patterning and a full-red pigmentation pattern (Fig. 6C). Hence, the purple patches of *P.* Ever Spring Prince ‘Plum’ was more likely due to reduced degradation of *PeMYB11* mRNA than to spot fusion.

### Solo-LTR of *HORT1* and differential miR858 expression are indispensable for generating fusion dark purple spots of harlequin *Phalaenopsis*

**Fig. 7:**
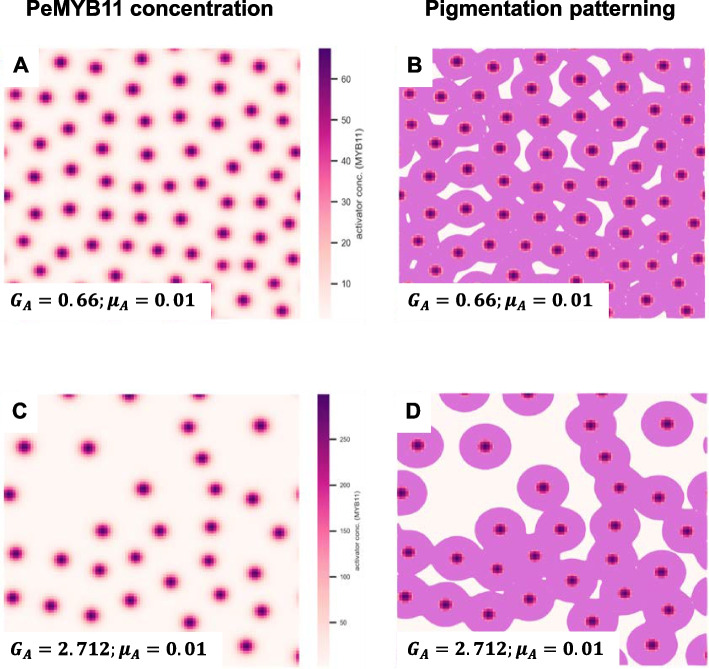
Simulation results of combination effects of insertion of solo-LTR of *HORT1* and reduced miR858 expression. **A** Simulated pattern of PeMYB11 with parameter values ${G}_{A}=0.66 \text{ and } {\mu }_{A}=0.01$. **B** is generated by plotting contour maps of (**A**) at the threshold of 2 $\mu M$. **C** Simulated pattern of PeMYB11 with parameter values ${G}_{A}=2.712 \text{ and } {\mu }_{A}=0.01$. **D** is generated by plotting contour maps of (**C**) at the threshold of 2 $\mu M$

The formation of dark purple patches of harlequin *Phalaenopsis* involved two factors: retrotransposon *HORT1* and miR858. These two factors influenced the pigmentation patterning in different ways, as confirmed by the numerical simulations above. We next evaluated the combination effect of these two factors. Simulation results showed an apparent fusion of spots (Fig. 7B, D) generated by plotting contour maps of (Fig. 7A, C). In addition, the region of each spot with the highest PeMYB11 level became larger (Supplementary Fig. 2B) than regions simulated with standard PeMYB11 degradation rate (Supplementary Fig. 2A), which was a similar pattern to the large dark purple area of *P*. Yushan Little Pearl. The similar characteristics between the outcomes of simulation and the phenotypes of *P.* Yushan Little Pearl suggest that reduced miR858 level in the purple regions of harlequin *Phalaenopsis* plays a role in promoting the fusion of large dark purple dots induced by the solo-LTR of *HORT1*.

### Spatially heterogeneous insertion of full-length HORT1 or solo-LTR of HORT1 in PeMYB11 promoter is a key factor in generating the dark purple patches of harlequin Phalaenopsis Yushan Little Pearl

In the diverse pigmentation patterning of harlequin *Phalaenopsis*, even though spot fusion is a common feature, the purple patches on the flowers have different patterning (Fig. 1): the purple patches may remain as round spots or be irregular shapes (Supplementary Fig. 3). Thus, in addition to both *HORT1* and miR858, other factors must shape different types of pigmentation patterning of harlequin *Phalaenopsis* and lead to varied color patterns.

**Fig. 8:**
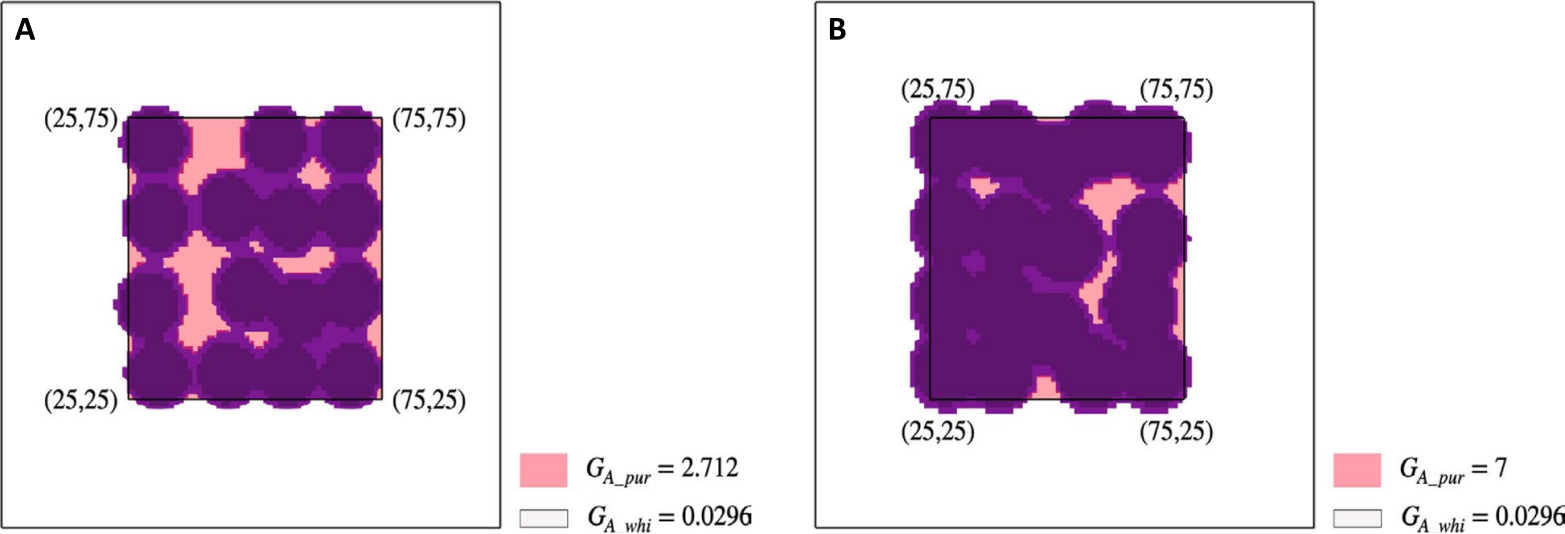
Rationale for the existence of full-length *HORT1* in purple regions of *P*. Yushan Little Pearl with piece-wise settings of ${G}_{A}$ values mimicking the heterogeneous distribution of full-length *HORT1* and solo-LTR of *HORT1.* **A** The heatmap of simulated PeMYB11 level is plotted with a basic threshold of 2 $\mu M$ and a relative threshold of 4 $\mu M$. Other parameter values are ${G}_{H}=0.12,{D}_{A}=0.01,{D}_{H}=0.5,{\mu }_{A}=0.01,{\mu }_{H}=0.03, {\rho }_{A}=0.01$. **B** The heatmap of simulated PeMYB11 level is plotted with a basic threshold of 2 $\mu M$ and a relative threshold of 5 $\mu M$. Other parameter values are ${G}_{H}=0.12,{D}_{A}=0.01,{D}_{H}=0.5,{\mu }_{A}=0.01,{\mu }_{H}=0.03, {\rho }_{A}=0.01$

**Fig. 9:**
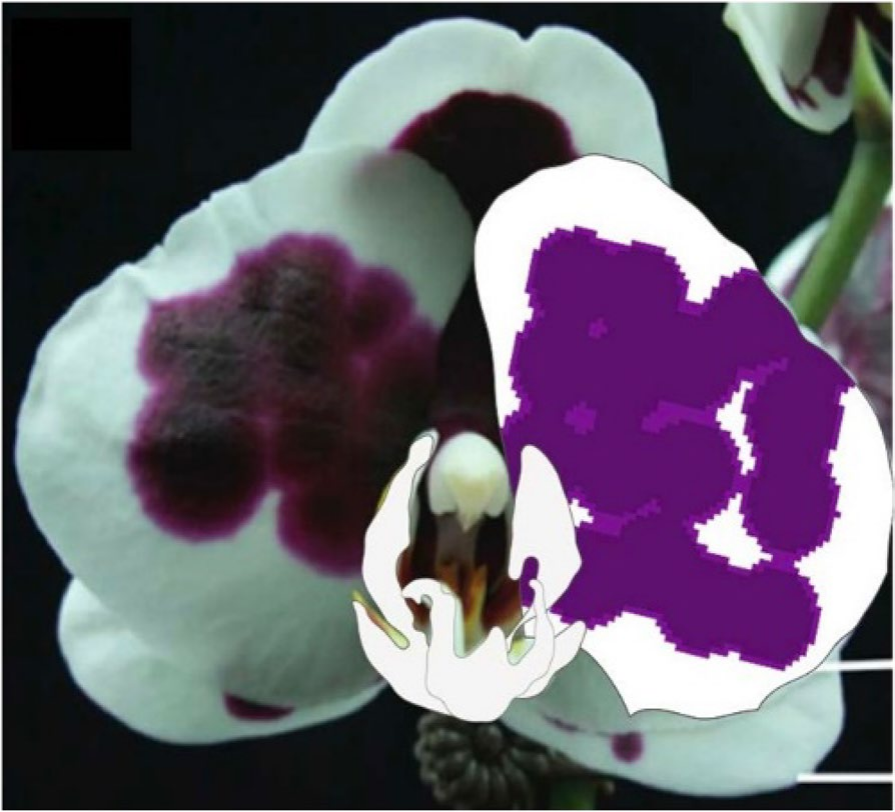
A comparison of the floral pattern of *P.* Yushan Little Pearl (left) and the simulated pattern (right). The simulation results recapitulated the characteristic pattern of harlequin *P.* Yushan Little Pearl, including a sharp separation between purple and white regions, large anthocyanin spots, and high levels of spot fusion. The simulation parameter values are as follows: ${{G}_{A\_pur}=7, {G}_{A\_whi}=0.0296, G}_{H}=0.12,{D}_{A}=0.01,{D}_{H}=0.5,{\mu }_{A}=0.01,{\mu }_{H}=0.03, {\rho }_{A}=0.01$

The identification of *HORT1* in the sepal/petal of *P.* Yushan Little Pearl has revealed the insertion of solo-LTR of *HORT1* together with full-length *HORT1* in the tissue of purple patches [2]. Simulation results indicated that some components of the 100 × 100 matrix, which were assigned with decreased transcription activity of *PeMYB11*, also showed anthocyanin accumulation (Fig. 8). Therefore, the existence of full-length *HORT1* in purple regions is reasonable, even though full-length *HORT1* reduces the transcription activity of *PeMYB11* [2]. The simulation results recapitulated the characteristic pattern of harlequin *P.* Yushan Little Pearl, including a sharp separation between purple and white regions, large anthocyanin spots, and high level of spot fusion (Fig. 9). Hence, the spatially heterogeneous insertion of *HORT1* and its structural variant are crucial factors for generating the patterns specific to *P.* Yushan Little Pearl.

## Discussion

In this study, we demonstrated that the PeMYB11-PeMYBx interaction model recapitulates the typical spotted patterning of harlequin *Phalaenopsis* orchids. The recapitulation suggests that the spatial distribution of anthocyanin gradually converges to a balance that generates periodic spots in *Phalaenopsis*. In harlequin *Phalaenopsis*, insertion of *HORT1* in the promoter of *PeMYB11* perturbed the transcriptional activity of *PeMYB11* [2]. Also, a reduced miR858 level perturbed the degradation of *PeMYB11* mRNA [5]. Our simulations indicated that variations in normal transcriptional activity and degradation rate of *PeMYB11* mRNA resulted in the extraordinary pigmentation patterning of harlequin *Phalaenopsis*.

In monkeyflowers (*Mimulus*), two MYB transcription factors, including an activator and an inhibitor, regulate the biosynthesis of each other and are related to the formation of the spotted patterning on flowers [9]. With numerical simulations, Ding et al. (2020) demonstrated that the interaction between two MYB transcription factors is critical for generating the spotted patterning [9]. Inspired by the previous study, we used the Gierer-Meinhardt model to assess the formation of spotted pigmentation patterning in harlequin *Phalaenopsis*. The PeMYB11-PeMYBx interaction model was a simplified and idealized assumption, but it helped retain critical features from a large amount of published experimental data related to the spotted patterning formation. With this concise biological model, we could clarify the main contributions of *HORT1* and miR858 to the exotic patterns of harlequin *Phalaenopsis*.

Bicolor floral phenotypes are unique in nature. A comprehensive picture of the underlying mechanisms is still under investigation. In fact, bicolor floral traits are related to the spatially differential regulation of anthocyanin biosynthesis genes such as the miR828/MYB12 module in Asiatic hybrid lily and the miR858/PeMYB11 module in *Phalaenopsis* ‘Panda’ [5, 13]. However, the reason for the existence of spatially differential expression of miRNAs is not clear. Actually, because of the diffusivity of miRNAs and the clear and balanced property of miRNAs [14, 15], identifying the source of miRNA is of primary concern. In *P.* ‘Panda’, the expression of miR858 is significantly enriched in the white regions where only full-length *HORT1* is identified. Moreover, miRNA can be derived from transposons [16]. Therefore, clarifying the interactions between *HORT1* and miR858 may provide a deeper understanding of the coloring mechanisms of bicolor flowers in harlequin *Phalaenopsis*.

Transposon/retrotransposon insertion is involved in the flower coloring mechanisms of bicolor flowers in plants [17]. Because of the “jumping traits” of transposons, transposition events may not coincide in every cell of all floral organs, which leads to irregular chimeric color regions on flowers. The retrotransposon *HORT1* has been identified to cause high anthocyanin accumulation in harlequin *Phalaenopsis*, so it is a potential factor in diverse pigmentation patterning [2]. Our study has shown that insertion of the solo-LTR of *HORT1* in the *PeMYB11* promoter is the key factor in generating large anthocyanin spots. In addition, *HORT1* is a non-autonomous retrotransposon because it lacks reverse transcriptase in its coding sequences (Supplementary Fig. 4). Therefore, instead of the “jumping traits” of retrotransposons, remnant solo-LTR of *HORT1* caused by random homologous unequal recombination of *HORT1* in individual cells of floral organs could explain the spatially heterogeneous insertion of *HORT1* and the diverse pigmentation patterning of harlequin *Phalaenopsis*.

According to the instability condition of the reaction–diffusion system, small random perturbations of the initial concentration of morphogens is essential for generating the Turing pattern [6]. In other words, if two initial concentrations of morphogens are slightly different, the patterns will not be the same. Consequently, two flowers on the same spike of one plant can have different pigmentation patterns. In fact, this is true for harlequin *Phalaenopsis* orchid flowers (Supplementary Fig. 5). In harlequin *Phalaenopsis* orchids, random spot fusion may result from the synergistic effects of the instability condition of the reaction–diffusion system and random homologous unequal recombination of *HORT1*.

Mathematical modeling in ornamental horticultural research has greatly advanced in recent years. Ringham et al., (2021) have generated pigmentation patterns directly on geometric models of flowers with various combinations of main mathematical models of morphogenesis [18]. These include vascular patterning, positional information, reaction–diffusion, and random pattern generation [18]. Their incorporation of these models demonstrate a wide array of the flower pigmentation patterns in nature, such as *Viola tricolor* (pansy), *Aquilegia* sp. (columbine), *Cypripedium reginae*, *Onosma alborosea*, *Malus domestica* (apple), *Oxalis adenophylla*, *Plumeria alba* (frangipani), *Gaillardia aristata*, *Wurmbea elatior*, *Galearis rotundifolia*, *Fritillaria meleagris* (checkered lily), and *Antirrhinum majus* (snapdragon) [18].

In the present study, we showed that mathematical modeling and simulation were efficient and systematic approaches to pinpoint hypotheses that were congruent to novel experimental discoveries. Mathematical modeling allows for constructing a well-grounded framework for research and also serves as the backbone for experimental designs. Therefore, it can be used to point out the correct direction for further research and avoid going in wrong directions. Mathematical modeling also contributes to the identification of critical molecular systems in complex biological processes [19]. It simplifies sophisticated problems and provides a new perspective to challenge established concepts. In the omics era, generating big data by using high-throughput sequencing and transcriptomics is common. The wealth of data can improve the precision of established mathematical models and assist in parameter decisions for the models [20].

## Conclusion

Mathematical modeling is a new and inevitable trend for studying complex biological processes. The mathematical model we developed substantiates the interactions among various factors including *HORT1*, solo-LTR, *PeMYB11*, *PeMYBx*, and miR858 that affect anthocyanin formation and accumulation in the harlequin *Phalaenopsis* orchids. This model holds great promise for developing efficient and innovative approaches to breeding the harlequin *Phalaenopsis* orchids.

## Materials and methods

### Construction of the PeMYB11-PeMYBx interaction model and its interplay with important factors

The construction of the PeMYB11-PeMYBx interaction model was based on experimental data (2,8). Notably, instead of representing a gene or a protein, “PeMYB11” and “PeMYBx” indicate systems of biological processes including transcriptional, post-transcriptional, and translational stages. The PeMYB11-PeMYBx interaction model comprises the following interaction rules: 1) PeMYB11 is self-activated, 2) PeMYB11 activates the biosynthesis of PeMYBx and 3) PeMYBx inhibits the biosynthesis of PeMYB11. We studied the direct effects of *HORT1* on the pigmentation patterning of harlequin *Phalaenopsis* by adjusting the self-activation rate of the activator reflecting the changes in transcription level of *PeMYB11*. In contrast, we studied the effect of miR858 on the pigmentation patterning by adjusting the degradation rate of the activator reflecting the changes in post-transcriptional level of *PeMYB11*. In addition to molecular factors such as *HORT1* and miR858, the spatial distribution of full-length *HORT1* and its solo-LTR throughout the petal/sepal tissue is a factor in the effect of the pigmentation patterning.

### Mathematical description of the PeMYB11-PeMYBx interaction model

**Fig. 10:**
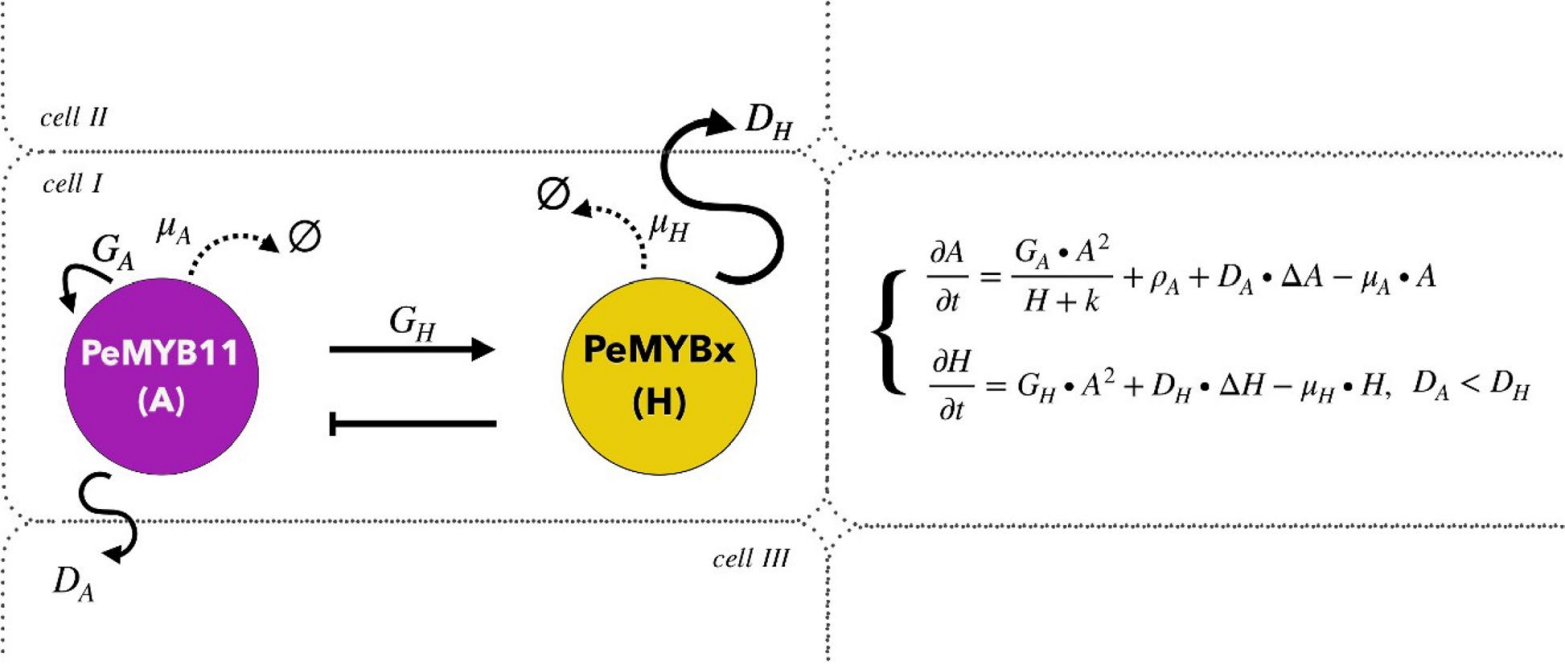
Mathematical description of the PeMYB11-PeMYBx interaction model. PeMYB11 is the activator of the reaction–diffusion system, and PeMYBx is the inhibitor of the system. PeMYB11 self-activates with the self-activation potency* G*_*A*_ value, and it also activates the biosynthesis of PeMYBx with the activation potency *G*_*H*_ value. PeMYBx inhibits the biosynthesis of PeMYB11. The activator and the inhibitor degrade with degradation rates ${\mu }_{A}$ and ${\mu }_{H}$, respectively. The activator diffuses with diffusivity *D*_*A*_, and the inhibitor diffuses with diffusivity *D*_*H*_. The value of *D*_*A*_ must be smaller than *D*_*H*_ for patterns to form. In the PeMYB11-PeMYBx interaction model, $\varnothing$ is the symbol for empty

Let $A(t,\mathbf{r}), H(t,\mathbf{r})$ denote the concentration of the activator of PeMYB11 and the inhibitor of PeMYBx at time t and location **r**, respectively, in the interaction model. The units of $A$ and $H$ are $\mu M.$ According to the interaction model (Fig. 10), we derived the following equations for the computational domain $\left(t,\mathbf{r}\right)\in \left[0,T\right]\times {\left[0, L\right]}^{2}$:

$$\left\{\begin{array}{c}\frac{\partial A}{\partial t}=\frac{{G}_{A}\bullet {A}^{2}}{H+k}+{\rho }_{A}+{D}_{A}\bullet \Delta A-{\mu }_{A}\bullet A,\\ \frac{\partial H}{\partial t}={G}_{H}\bullet {A}^{2}+{D}_{H}\bullet \Delta H-{\mu }_{H}\bullet H,\end{array}\right.$$

where $T$ is the stopping time, $L$ is the side length of the computational square and $\Delta$ is the Laplacian operator in space. At each computation grid, the initial concentration of $A(0,\mathbf{r})$ is randomly assigned with values from 0 to 1 (9). The initial concentration of $H\left(0,\mathbf{r}\right) \text{is }0$ because the biosynthesis of PeMYBx is activated by PeMYB11. The boundary condition is assumed to be the periodic boundary condition. The meaning of the coefficients in the equations is as follows: ${\text{G}}_{\text{A}}$ represents the potency of self-activation of ${A}^{2}$; ${\text{G}}_{\text{H}}$ represents the potency of activation of ${\text{H}}$ activated by ${A}^{2}$; ${\text{D}}_{\text{A}}$ and ${D}_{H}$ represent the diffusion rate of $\text{A and H}$, respectively; and $\mu_A$ and ${\mu }_{H}$ represent the degradation rate of A and H respectively. ${\text{D}}_{\text{H}}$ must be larger than ${\text{D}}_{\text{A}}$ (4).

### Computational simulations of the PeMYB11-PeMYBx interaction model: simulation of typical spotted patterning

We wrote the script for performing 2-D numerical simulations in Python to generate the spotted patterning. Without loss of generality, we set $L \text{to} 100$ and used the uniform grid in space, that is, all integer grid points $\left(i,j\right), 1 \le i, j \le 100,$ in the computational square for two concentrations, $A$ and $H$. For the initial setting, random values were assigned to $A$, the concentration of PeMYB11. The initial concentration of PeMYBx, $H$, was a zero matrix. The value of each parameter in the model was the same as in Ding et al*.* (2020) ${(D}_{A}=0.01,{D}_{H}=0.5,{\mu }_{A}=0.03,{\mu }_{H}=0.03,{G}_{A}=0.08,{G}_{H}=0.12,{\rho }_{A}=0.01, \text{and} k=0.001)$, which is widely accepted from previous studies. We used rdsolver, a Python package for solving reaction–diffusion systems (*v0.1.5*), to simulate the dynamic behavior of the discrete system of the PeMYB11-PeMYBx interaction model. We chose the rdsolver package because it uses variable time step implicit/explicit (VSIMEX) time stepping to attain better computer efficiency for solving the equations. With rdsolver, the concentrations of both PeMYB11 and PeMYBx were solved. In addition, to validate the correctness of the simulation results, comparison simulations were computed with Virtual Cell software by modifying the model constructed by Ding et al. (2020). Virtual Cell is a mature and free software for modeling and simulating biological systems (10). Nevertheless, Virtual Cell does not allow for considering piece-wise ${G}_{A}$ values. Thus, in this study, we designed a new script to apply piece-wise ${G}_{A}$ values.

### Increased *PeMYB11* transcription activity induced by the solo-LTR of *HORT1*

All parameter values were the same as those in the numerical simulation for typical spotted pigmentation patterning, except for adjustment of the self-activation potency ${G}_{A}$ value. The adjustment of the *G*_*A*_ value was initially derived from the experimental data for promoter activity with dual luciferase assay (2). To resemble the enhanced transcription activity induced by insertion of the solo-LTR of *HORT1* in the promoter of *PeMYB11*, ${G}_{A}$ was adjusted to about twofold of the standard *G*_*A*_ value (= 0.08) and eventually equaled 0.1632. In addition, harlequin *Phalaenopsis* cultivars are tetraploid plants (11), so we also considered another value for *G*_*A*_ (0.1632 $\text{x}$ 4 $\approx$ 0.66) for tetraploid plants. Furthermore, the expression of *PeMYB11* is 33.9-fold higher in the purple region of* P*. Yushan Little Pearl than in *P*. OX Red Shoe ‘OX1408’ (2). The transcription activity of *PeMYB11* is enhanced by insertion of the solo-LTR of *HORT1* but not the full-length *HORT1*, so the *G*_*A*_ value was adjusted to 2.712 (= 33.9 $\text{x}$ 0.08) to reflect the enhanced *PeMYB11* transcription activity in the purple regions of* P*. Yushan Little Pearl. In addition, we also considered the ${G}_{A}$ values of 7 and 70 in later simulations for observing the rate of changes of spot sizes, approximately tenfold and 100-fold increases of the 0.66 value, respectively, in tetraploid plants.

### Reduced degradation rate of *PeMYB11* caused by reduced miR858 expression

All parameters were the same as those in the numerical simulation for typical spotted pigmentation patterning, except for adjustment of the degradation rate ${\mu }_{A}$. To resemble the reduction in miR858 expression, the value of ${\mu }_{A}$ was adjusted to less than 0.5-fold of 0.03 (standard ${\mu }_{A}$) according to the experimental results of quantitative PCR of miR858 (8). The number 0.01 was selected as the lowered value of ${\mu }_{A}$. To assess the combination effects of the solo-LTR of *HORT1* and miR858, we used only the parameters ${G}_{A}$ and ${\mu }_{A}$ without affecting other parameters, and the adjustment was based on the two independent cases for solo-LTR of *HORT1* and miR858.

### Comprehensive consideration of the heterogeneous spatial distribution of full-length *HORT1* and solo-LTR of *HORT1*

**Fig. 11:**
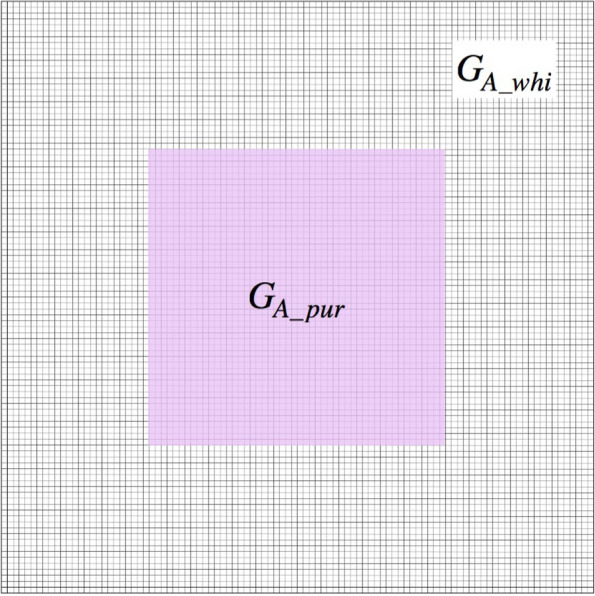
The rule for assigning piece-wise ${G}_{A}$ for simulating the effect of heterogeneous transcription activity of *PeMYB11* in *P.* Yushan Little Pearl

Identification of both full-length *HORT1* and solo-LTR of *HORT1* in the flower of harlequin *P.* Yushan Little Pearl indicated the existence of heterogeneous transcription activity of *PeMYB11* in sepal/petal tissue. To simulate the effect of heterogeneous transcription activity of *PeMYB11*, we assigned piece-wise ${G}_{A}$ values to the computation grids. The assignment rule is illustrated in Fig. 11. The value of ${G}_{A\_pur}$ is larger than the standard value 0.08. The value of *G*_*A_whi*_ was assigned as 0.0296 by multiplying the standard *G*_*A*_ value (= 0.08) by 0.37 according to the experimental data for the effect of inserting the full-length *HORT1* in promoter activity by using dual luciferase assay (2). The transcription of *PeMYB11* is strongly inhibited in the white region. Consequently, even though harlequin *Phalaenopsis* cultivars are tetraploid plants, we used only one-fold of the value 0.0296 (= 0.08 × 0.37) to resemble the inhibition effect of insertion of the full-length *HORT1* on *PeMYB11* transcription.

### Visualization of the pattern of simulated PeMYB11 and PeMYBx levels

To visualize the patterns, the 2-D solutions of PeMYB11 and PeMYBx were colored by plotting the heatmaps of the 100 × 100 matrix using Matplotlib and seaborn packages. When each parameter assigned to each computation grid was uniform, we set a threshold of 2 $\upmu M$ for visualizing the pattern. When piece-wise ${G}_{A}$ was assigned, an additional threshold $T$ > 2 $\upmu M$ was defined for a deep coloring layer to mimic the floral-color gradient filling of flowers of *P.* Yushan Little Pearl. To obtain a consistent color-filling rule over different pairs of ${G}_{A\_pur}$ and ${G}_{A\_whi}$ values, $T$ was defined as the ${}^{{G}_{A\_whi}}\!\left/ \!{}_{{G}_{A\_pur}}\right.$ proportion of the maximum PeMYB11 level.

### Supplementary Information

Supplementary Material 1.

## Data Availability

The code for pattern simulation is available at https://figshare.com/s/0bc3fd0532a1bb336edb.

## Abbreviations

HORT1: *Harlequin orchid retrotransposon 1*
LTR: Long terminal repeat
MBW: R2R3-MYB-bZIP-WD40
miR156: MicroRNA156
