# Correction: Perturbation of periodic spot-generation balance leads to diversified pigmentation patterning of harlequin Phalaenopsis orchids: *in silico* prediction

**DOI:** 10.1186/s12870-024-05475-w

**Published:** 2024-08-17

**Authors:** Ti-Wen Lu, Wen-Huei Chen, Pao-Yang Chen, Yu-Chen Shu, Hong-Hwa Chen

**Affiliations:** 1 Department of Life Sciences, National Cheng Kung University, Tainan, 701 Taiwan; 2 Orchid Research and Development Center, National Cheng Kung University, Tainan, 701 Taiwan; 3 Institute of Plant and Microbial Biology, Academia Sinica, Taipei, 115 Taiwan; 4 Department of Mathematics, National Cheng Kung University, Tainan, 701 Taiwan

**Correction: BMC Plant Biol 24**,** 681 (2024)**

10.1186/s12870-024-05305-z

Following publication of the original article [1], the authors identified errors in the numbering of figures due to the movement of Materials and Methods section at the end of the article. The current Figs. 2 and 3 need to be switched to the end of the article to be Figs. 10 and 11, and the correct Figs. 2, 3, 4, 5, 6, 7, 8 and 9 are the current Figs. 4, 5, 6, 7, 8, 9, 10 and 11.

The original article [1] has been corrected.
