# Supplementary material for: Perturbation of periodic spot-generation balance leads to diversified pigmentation patterning of harlequin Phalaenopsis orchids: in silico prediction
Source: BMC Plant Biol. 2024 Jul 18;24:681. doi: 10.1186/s12870-024-05305-z (PMC11330024; doi:10.1186/s12870-024-05305-z)
Supplement: Supplementary file 1 — Supplementary Material 1. [file 12870_2024_5305_MOESM1_ESM.pdf]

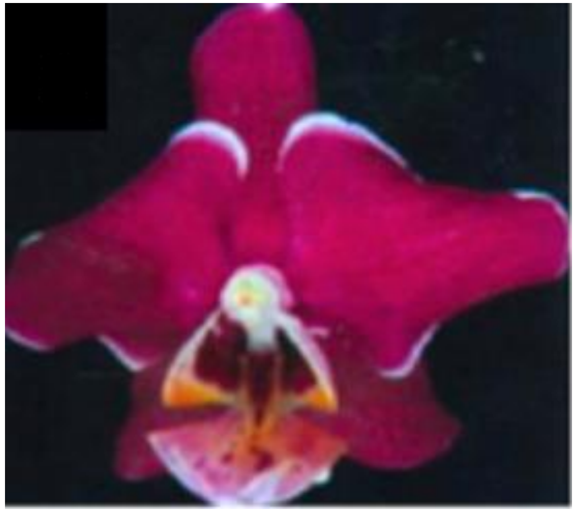

Lu *et al*., Supplementary Figure 1. *Phalaenopsis* Ever-spring Prince 'Plum‘. has large purple patches. The anthocyanin level in the purple patches of *P.* Ever Spring Prince ‘Plum’ are uniformly distributed throughout the flower.

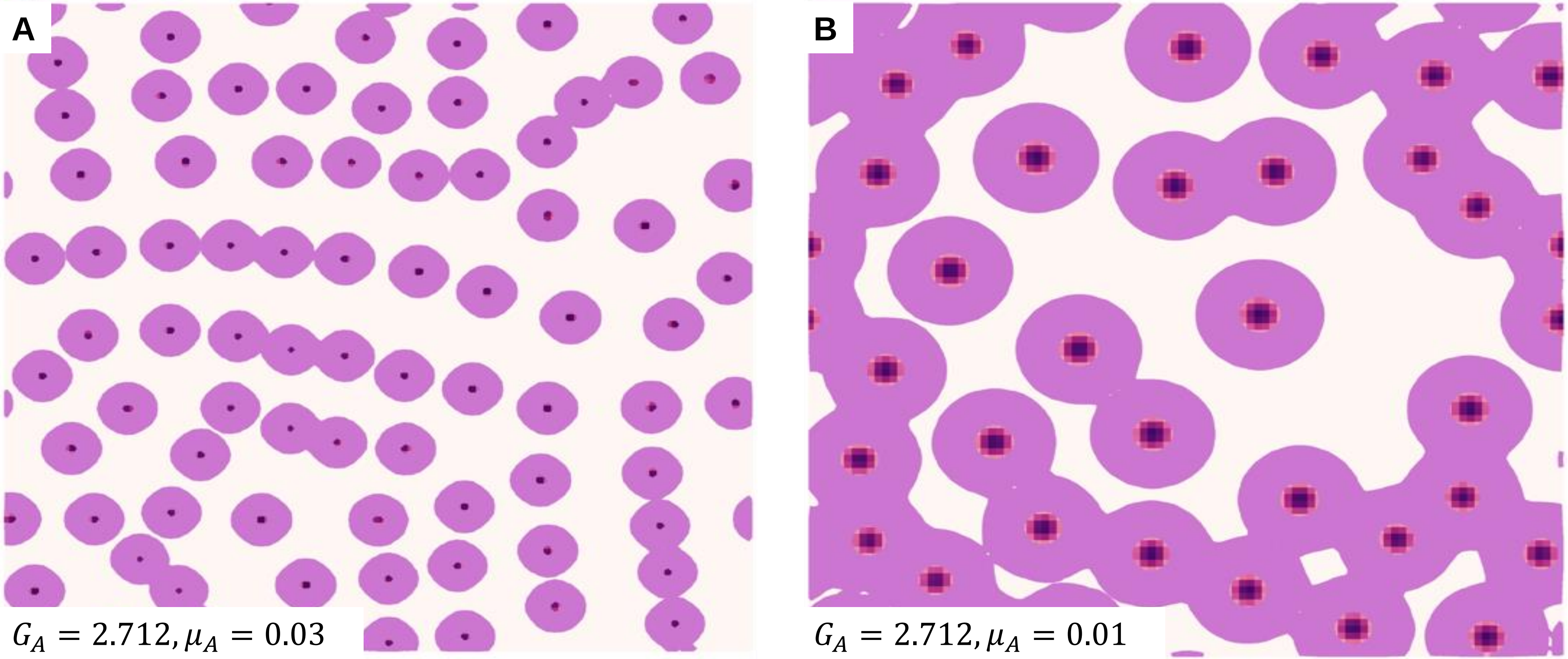

Lu *et al*., Supplementary Figure 2. Combinatorial effects of solo-LTR of *HORT1* and reduced miR858 expression result in apparent spot fusion. (a) The simulation result where only the effects of solo-LTR of *HORT1* was considered. (b) The simulation result where combinatorial effects of solo-LTR of *HORT1* and miR858 expression were considered.

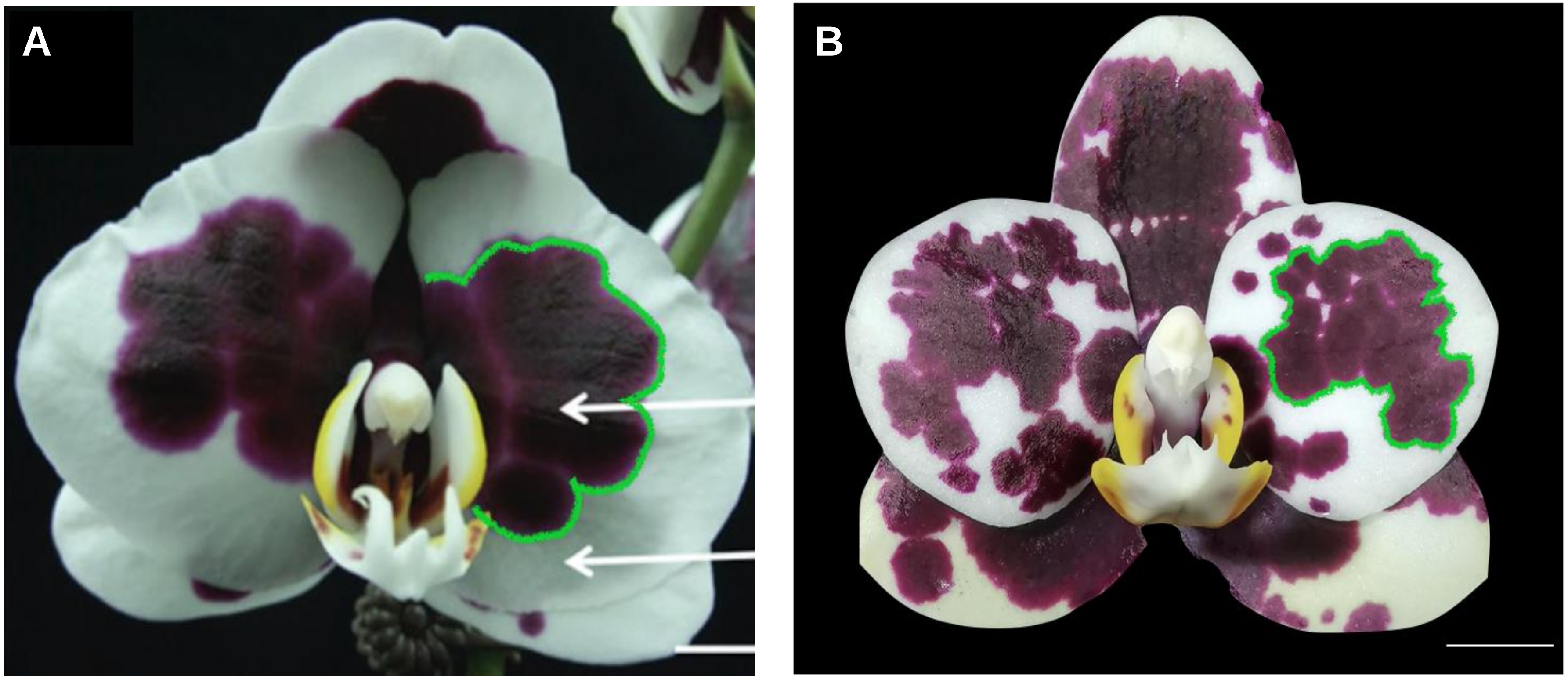

Lu *et al*., Supplementary Figure 3. Different types of outlines of the purple patches of harlequin *Phalaenopsis* (a) *Phalaenopsis* Yushan little pearl (b) *Phalaenopsis* Golden Peoker ‘Black’.

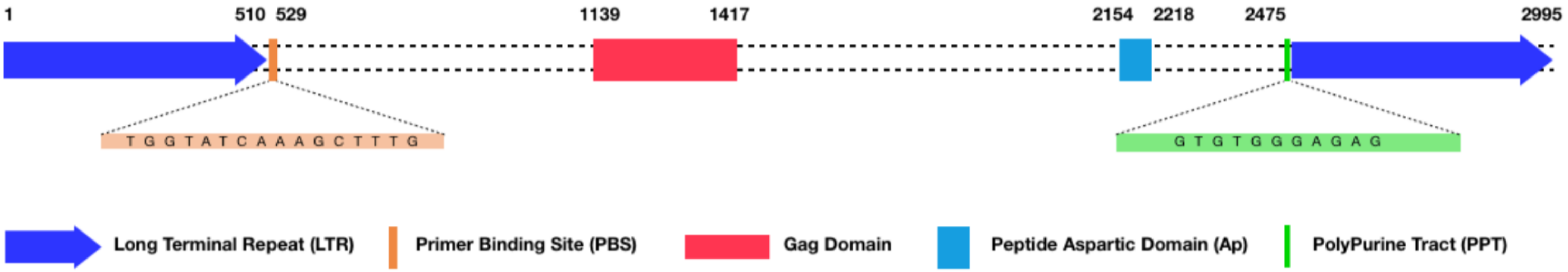

Lu *et al*., Supplementary Figure 4. *HORT1* is a non-autonomous retrotransposon. It has two LTRs (blue color), but lacks reverse transcriptase in its coding sequence. The coding region only codes for the Gag domain (red color).

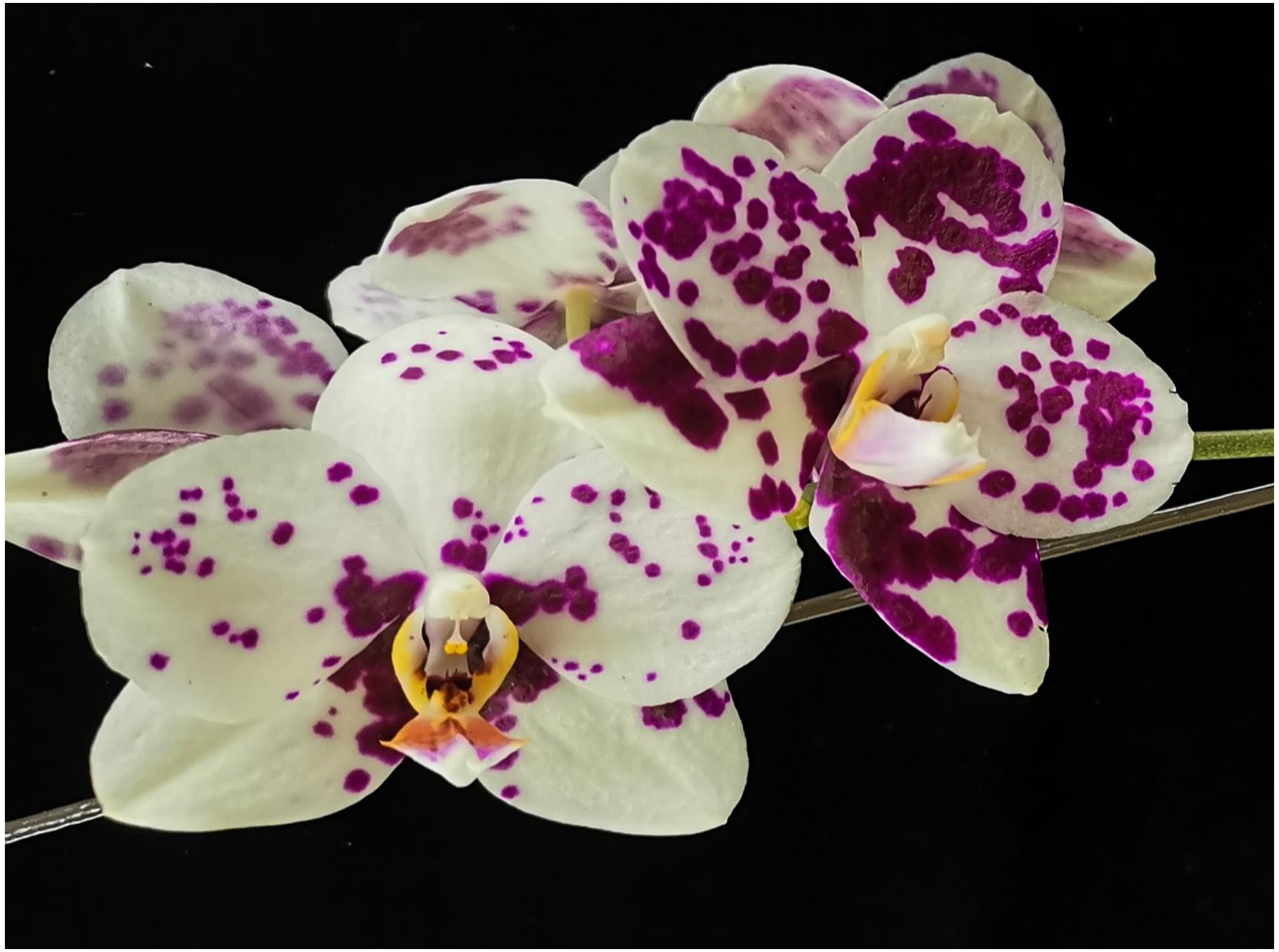

Lu *et al*., Supplementary Figure 5. Diverse pigmentation patterning on different flowers blooming on the same harlequin *Phalaenopsis* Golden Peoker ‘Black’.
